# Exploring Thermal Stability, Vibrational Properties, and Biological Assessments of Dichloro(l-histidine)copper(II): A Combined Theoretical and Experimental Study

**DOI:** 10.1021/acsomega.4c05029

**Published:** 2024-10-16

**Authors:** Kamila R. Abreu, Jailton R. Viana, João G. Oliveira Neto, Tatielle G. Dias, Aramys S. Reis, Mateus R. Lage, Luzeli M. da Silva, Francisco F. de Sousa, Adenilson O. dos Santos

**Affiliations:** † Center for Sciences of Imperatriz, Federal University of Maranhao (UFMA), 65900-410 Imperatriz, MA, Brazil; ‡ Institute of Exact and Natural Sciences, Federal University of Para (UFPA), 66075-110 Belem, PA, Brazil

## Abstract

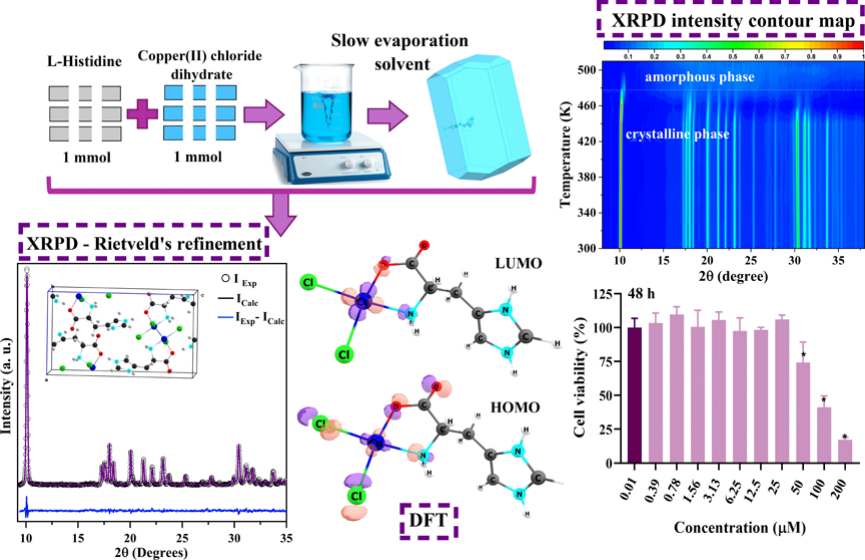

Dichloro(l-histidine)copper(II) crystal ([Cu(l-His)Cl_2_] complex) was obtained by the slow evaporation method and characterized concerning its thermal stability, phase transformations, and electronic and vibrational properties. X-ray diffraction (XRPD) confirmed that this complex crystallizes with an orthorhombic structure (*P*2_1_2_1_2_1_ space group). Thermal analyses (TG and DTA) demonstrate stability from ambient temperature up to 460 K, followed by a phase transition from the orthorhombic structure to the amorphous form around 465 K, as confirmed by temperature-dependent XRPD studies. The active modes in Fourier transform infrared (FT-IR) and Raman spectroscopy spectra were suitably assigned via density functional theory (DFT) calculations. Additionally, Hirshfeld surface analysis uncovered the prominence of Cl···H, O···H, and H···H interactions as the primary intermolecular forces within the crystal structure. The antimicrobial activity of the [Cu(l-His)Cl_2_] complex was investigated, demonstrating significant efficacy against Gram-positive bacteria (*Staphylococcus aureus*), Gram-negative bacteria (*Pseudomonas aeruginosa*), and fungi (*Candida albicans*). The minimum inhibitory concentration and cell viability tests showed that the complex inhibits the growth of *S. aureus* bacteria at a concentration of 1.5 μM without causing damage to the human cell line. The pharmacokinetic parameters corroborate the other tested parameters and highlight the [Cu(l-His)Cl_2_] complex as a promising alternative for future clinical trials and medicinal applications. The alignment of the pharmacokinetic parameters with other tested criteria highlights the potential of the [Cu(l-His)Cl_2_] complex as a promising candidate for future clinical studies.

## 1 Introduction

In recent years, the search for coordination compounds for application in biological activity has intensified, driven by factors such as cost-effective manufacturing processes and optimal performance.^1,2^ In this context, a specific area of heightened interest is synthesizing and characterizing novel complexes.^3−6^ Complexes play a crucial role in many fields of chemistry, from industrial applications to biochemistry and catalysis. The formation of metal complexes occurs due to the ability of a metal (atom or ion) to accommodate pairs of electrons donated by the ligands to form coordinated covalent bonds.

Copper (Cu) is an essential element related to the functioning of several enzymes and proteins involved in energy metabolism and respiration and protecting cells against damage caused by free radicals through antioxidant action. As an endogenous metallic element, copper exhibits fewer toxic adverse side effects than exogenous metals such as platinum.^7^ These properties have been explored in developing new copper complexes with therapeutic potential for treating several diseases.^8−10^

Specifically, developing Cu^2+^-based complexes with antimicrobial activity is a relevant subject. Recent studies have shown that copper complexes exhibited enhanced bactericidal effects by inducing oxygen-free radicals and cleavage effects.^11−14^ Although copper has long been recognized as a broad-spectrum antimicrobial agent, there are still concerns about its application due to its toxicity. On the other hand, the emergence of bacteria resistant to classical antibiotics and the scarcity of new compounds in development has increased interest in copper as an antimicrobial agent. In this regard, using copper in complexes, when appropriately formulated, can provide effective antimicrobial activity while minimizing toxicity concerns.^15,16^

Amino acids and their derivatives emerge as promising candidates for ligands in complexes due to their coordination properties and biological importance. Incorporating amino acid ligands into complexes can significantly impact pharmacological aspects, including solubility, bioavailability, and transport mechanisms, enhancing therapeutic efficacy and minimizing potential side effects.^17^

Among amino acids, l-histidine (l-His) is an essential amino acid that acts as a ligand species for Cu^2+^ ions in many copper proteins. l-His offers three distinct binding sites: the carboxylic, imidazole, and primary amine groups, affording versatile coordination possibilities as a mono-, bi-, or tridentate ligand.^18−21^ Studies of Cu^2+^ complexes containing the amino acid l-His as a ligand are of great interest due to their biological relevance since l-His is commonly found in metalloproteins.^22−24^ Furthermore, these complexes can present a great structural variety with different mechanisms of action depending on how the l-His interacts with Cu^2+^.^25−27^

In particular, the dichloro(l-histidine)copper(II) complex, herein termed the [Cu(l-His)Cl_2_] complex, is an interesting system for thorough investigation. As reported by Colyvas and coauthors,^28^ the [Cu(l-His)Cl_2_] complex comprises molecular chains with the carboxy oxygen atom, the amino nitrogen atom, and the two chlorine atoms forming an approximate square plane about the copper atom. Two bridging chlorine atoms from adjacent molecules contribute to a distorted octahedral geometry. These chains are interconnected via extensive hydrogen bonding. Li Jianmin reported electronic and photoacoustic absorption spectra measurements, along with analysis using ligand field theory (LFT) and the radial wave function of nonfree copper(II) for the [Cu(l-His)Cl_2_] complex, suggesting the presence of significant magnetic exchange interaction in this material.^29^ Although these previous studies have provided insights into the structural and optical properties of the [Cu(l-His)Cl_2_] complex, there is still a lack of information about electronic, vibrational, and thermal properties, as well as theoretical studies to validate data. Moreover, no biological tests have been reported to investigate the potential of the [Cu(l-His)Cl_2_] complex for biological applications.

This study reports the [Cu(l-His)Cl_2_] complex synthesis using the slow evaporation method and examines its structural, thermal, spectroscopic, and biological properties. Vibrational mode assignments were conducted through quantum chemical calculations based on density functional theory (DFT.) The *in silico* pharmacokinetic parameters of the [Cu(l-His)Cl_2_] complex were obtained. Biological assays were conducted to evaluate the complex activity against Gram-positive bacteria (*Staphylococcus aureus*), Gram-negative bacteria (*Pseudomonas aeruginosa*), fungi (*Candida albicans*), and the cell viability of normal lung fibroblasts (GM07492A).

## 2 Materials and Methods

### 2.1 Crystal Growth

The [Cu(l-His)Cl_2_] crystals were grown by using the slow solvent evaporation method. A solution containing 1 mmol of copper II chloride dihydrate (Sigma-Aldrich, purity >98.9%) was prepared in 10 mL of deionized water. This solution was then added to a second solution composed of 1 mmol of l-His (Sigma-Aldrich, purity >99%), 40 mL of methanol (purity 98%), and 10 mL of deionized water. The two solutions were mixed and maintained under magnetic stirring at 300 rpm for 2 h at 300 K temperature. Subsequently, the final solution with pH 7.1 was filtered and stored under 300 K. After 5 weeks, blue crystals were obtained.

**Scheme 1:**
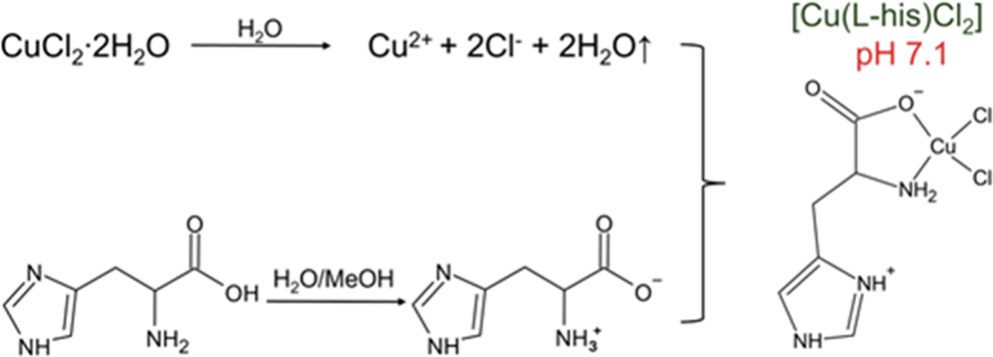
Synthesis Route of the [Cu(l-Hist)Cl_2_] Complex

Scheme 1 illustrates the formation of the complex from the chemical reaction that occurs between the ions formed from the reactants. As demonstrated, upon dissolving CuCl_2_·2H_2_O in water, Cu^2+^ and two Cl^–^ ions are released in solution at an acidic pH (approximately 3.7). In contrast, the dissolution of l-His in a mixed solution of water and methanol (MeOH) occurs in a basic medium (pH around 7.6). In this basic environment, l-His exists as a zwitterion, with the amino group protonated (NH_3_^+^) and the carboxyl group (COOH) deprotonated, forming a carboxylate (R-COO^–^). When the resulting solutions are mixed, the Cl^–^ anions and the l-His ligand, through an oxygen atom of the carboxylate group (COO^–^) and the nitrogen atom of the amine (NH_2_), coordinate to the Cu^2+^ ion. This coordination forms the complex with a number of coordinations (NC) of 4 and a distorted square planar geometry. In the resultant mixture, the second nitrogen atom of the five-membered ring protonates and the resulting total charge of the complex is zero.

### 2.2 Spectroscopy in the Ultraviolet–Visible (UV–vis) Region

The Thermo Scientific Evolution 220 UV–vis spectrophotometer was employed to register spectra within the 400–900 nm range. The instrument, featuring a double-beam design, was equipped with a deuterium lamp and utilized quartz cuvettes with an optical path length of 0.1 cm.

### 2.3 X-ray Powder Diffraction (XRPD)

X-ray powder diffraction (XRPD) measurements were performed in an Empyrean diffractometer (PANalytical), using Cu Kα radiation (λ = 1.5418 Å), operating at 40 kV/40 mA. The diffraction patterns were collected from 8 to 50° (2θ) with a step size of 0.02° and a counting time of 50 s. The XRPD pattern was analyzed by Rietveld’s refinement using the GSAS program.^30,31^ The structure was refined based on the unit cell parameters reported in the literature.^28^ The new data has been submitted to the Cambridge Crystallographic Data Center (CCDC), from the crystallographic information file (.cif) generated under the code CCDC 2373288 (see the Supporting Information). This data can be obtained free of charge at https://www.ccdc.cam.ac.uk.

Temperature-dependent measurements ranging from 300 to 465 K were conducted under vacuum conditions by employing an Anton-Paar TTK 450 temperature chamber coupled with the diffractometer.

### 2.4 Computational Technique

Geometry optimization and vibrational frequency calculations were performed using Gaussian 16 software.^32^ The calculations were developed using the DFT functional PBE1PBE with the basis set 6-311++G(d,p) for Cl, C, N, and O atoms, while the Stuttgart-Dresden pseudopotential and SDD basis set were used for the Cu atom.^33−36^ From the calculations, we confirmed that the optimized geometry obtained corresponds to a minimum in the potential energy surface since all vibrational frequencies calculated are positive. A scale factor of 0.959 was applied to adjust the vibrational frequencies obtained, ensuring better alignment between theoretical and experimental data.^37^ The vibrational modes were analyzed and assigned with vibrational mode automatic relevance determination (VMARD) using the VibAnalysis program.^38^

In addition, the variation of Gibbs free energy (*G*^298^), enthalpy (*H*), and total electronic energy corrected with zero-point vibrational energy (*E*_ZPVE_) associated with the complexation were calculated using the equation:
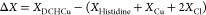
1where *X* represents *G*^298^, *H*, or *E*_ZPVE_.

From the study of frontier molecular orbital HOMO (highest occupied molecular orbital) and LUMO (lowest unoccupied molecular orbital) data, we calculated the HOMO–LUMO gap (HLG) and chemical reactivity descriptors for the [Cu(l-His)Cl_2_] complex. The descriptors were calculated based on eqs 2–5, corresponding to chemical potential (μ), electronegativity (χ), hardness (η), and softness (σ), respectively, as follows:
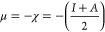
2
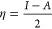
3
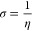
4
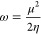
5In these equations, the ionization energy (*I*) and the electronic affinity of the fundamental state (*A*) are taken as the negative of HOMO energy and the negative of LUMO energy, respectively, in accordance with the Koopmans’ theorem.^39,40^

The Hirshfeld surfaces (HS) and two-dimensional (2D) fingerprint graphics were obtained using the CrystalExplorer 17 software,^41,42^ exploring crystallographic data of the [Cu(l-His)Cl_2_] complex collected from the Cambridge Crystallographic Data Center (CCDC - reference code: 1113517).^28^ These analyses enable a detailed study of intermolecular interactions between chemical species belonging to the crystal. The surfaces were plotted as a function of the normalized distance (*d*_norm_) to provide three-dimensional (3D) representations of close contacts, defined in terms of distances from a given point on the surface to the nearest external (*d*_e_) and internal (*d*_i_) with a default color. The fingerprint graphics were plotted as a function of *d*_*e*_ and *d*_*i*_, showing specific atomic interactions.

### 2.5 Fourier Transform Infrared (FT-IR) Spectroscopy

Fourier transform infrared (FT-IR) spectrum was acquired by analyzing a powdered sample within the 4000–300 cm^–1^ range (mid-infrared) using attenuated total reflectance (ATR) mode on a Bruker Vertex 70 v spectrometer. The setup included an accessory setting A225/Q Platinum ATR and a detector setting RT-Dla TGS wide range MIR-FIR with a 6 mm aperture, allowing for the measurement of spectra with a resolution of 4 cm^–1^ and an average of 100 scans.

### 2.6 Raman Spectroscopy

The Raman spectrum was recorded within the 30–3600 cm^–1^ range utilizing a triple-grating spectrometer (Jobin-Yvon, T64000) coupled to a CCD detection system and a thermoelectric cooling system based on Peltier-cooled technology. A solid-state laser (λ = 532 nm) with power at 0.5 mW was used as an excitation source. The spectrum was obtained with a spectral resolution of 2 cm^–1^.

### 2.7 Thermal Analyses (TG, DTA, and DSC)

Thermogravimetry (TG) and differential thermal analysis (DTA) measurements were carried out simultaneously in a Shimadzu DTG-60 thermogravimetric analyzer. A sample of about 3.5 mg was uniformly distributed on the α-alumina crucible and heated from 300 to 1000 K at a heating rate of 10 K/min under a synthetic air atmosphere (100 mL/min).

Differential scanning calorimetry (DSC) analysis was performed using Shimadzu DSC 60 equipment calibrated with pure in standard (99.99%). In this analysis, 3.3 mg of the sample was uniformly distributed on the sealed aluminum crucible and heated from 300 to 503 K with a 100 K/min heating rate under a synthetic air atmosphere (100 mL/min).

### 2.8 Antimicrobial Activity

Bacterial strains of *Staphylococcus aureus* ATCC 25923 (Gram-positive) and *Pseudomonas aeruginosa* ATCC 27853 (Gram-negative), and the fungus strain *Candida albicans* ATCC 10231 (yeast) were employed to assess the antimicrobial activity of the complex. The bacterial strains were preserved on Mueller-Hinton Agar at 4 °C and cultured in Mueller-Hinton broth at 37 °C before each experiment. The yeast strain was stored on Sabouraud Dextrose Agar at 4 °C and cultured in Sabouraud Dextrose Broth at 37 °C before the experiments. Antimicrobial activity was evaluated using the microplate microdilution method following the guidelines provided by the Clinical and Laboratory Standards Institute.^43,44^

Initially, serial dilutions (1:1) were made from the [Cu(l-His)Cl_2_] complex (ranging from 200 to 0.39 μM) in sterile 96-well microplates using a specific broth for each type of microorganism. Subsequently, according to McFarland’s 0.5 scales,^45^ approximately 1.5 × 10^8^ CFU/mL of the bacteria or 10^6^ CFU/mL of the yeast were added using Mueller-Hinton broth for bacteria, and RPMI 1640 medium (Sigma-Aldrich) buffered with 0.165 M MOPS for yeast, in a total volume of 200 μL/well. The standard drugs Meropenem (261–0.50 μM) and fluconazole (209–0.41 μM) were used as positive controls for bacteria and fungi, respectively. The microplates were incubated at 37 °C for 24 h for bacteria and 48 h for yeast. The minimum inhibitory concentration (MIC) was defined as the lowest concentration that inhibited the visual growth of the microorganisms. The experiments were performed in triplicate.

### 2.9 Viability Assay in Human Cells

The human cell line GM07492A (normal lung fibroblasts) was used for this study. Cells (5 × 10^5^ cells/mL in 100 μL/well) were incubated in 96-well microplates with DMEM supplemented with 10% fetal bovine serum, 2 mM glutamine, 100 U/mL penicillin, and 100 μg/mL streptomycin at 310 K with 5% CO_2_. After 1 h of culture, for spreading and adhesion, the cells were treated with [Cu(l-His)Cl_2_] complex (200–0.39 mM) for 24 and 48 h, at 310 K with 5% CO_2_. The viability was evaluated using the 3-(4,5-dimethyl-2-thiazolyl)-2,5-diphenyl-2H-tetrazolium bromide (MTT) assay.^46^

At the end of incubation, the supernatant was removed and replaced with a fresh medium (100 μL) containing the dye MTT (0.5 mg/mL). Cell viability was quantified by the ability of live cells to reduce the yellow dye MTT to a purple formazan product by metabolically active cells. After 3 h, the plates were centrifuged, the MTT formazan product was dissolved in 100 μL of dimethyl sulfoxide (DMSO), and the absorbance was measured using a multiplate reader at 550 nm. The MIC was defined as the lowest concentration that statistically induced the death of cells. The data were analyzed using ANOVA with Dunnett multiple, with a reliability level of 5% (*p* < 0.05). The concentration of DMSO solvent used does not affect the antimicrobial activity of the complex against the tested microorganism strains.

### 2.10 ADME Analysis of Pharmacokinetic Parameters

In investigating the pharmacokinetics, we employed SwissADME,^47^ a freely available software tool developed and maintained by the Molecular Modeling Group of the Swiss Institute of Bioinformatics. This software facilitated the analysis of physicochemical characteristics, lipophilicity, water solubility, and drug parameters pertaining to the [Cu(l-His)Cl_2_] complex under study. The 2D structural model of the optimized complex was input into ChemAxon’s Marvin JS window for molecular sketching and subsequently converted into SMILES format. These results were then evaluated utilizing the established guidelines of Lipinski,^48^ Ghose,^49^ Veber,^50^ and Egan,^51^ which offer valuable insights into the pharmacokinetic properties of novel drugs.

## 3 Results and Discussion

### 3.1 Spectroscopy in the UV–Vis Region

**Figure 1:**
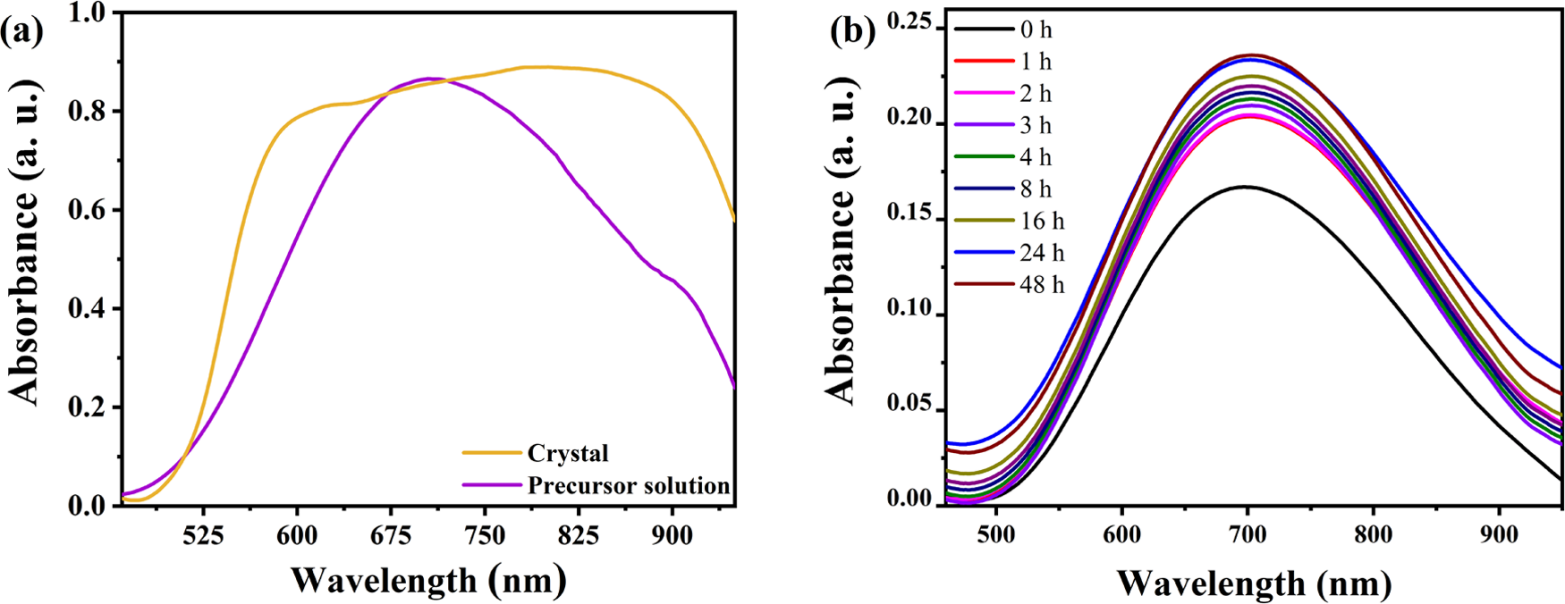
(a) UV–vis spectrum of the [Cu(l-His)Cl_2_] complex in the precursor liquid solution and in the solid (crystal) form. (b) UV–vis spectra as a function of time for the [Cu(l-His)Cl_2_] complex dissolved in an aqueous solution.

In Figure 1a, we can observe the absorption spectra in the UV–vis region for the complex in the precursor liquid solution and the solid (crystal) form. These spectra help us see the d–d type’s electronic transitions, which are usually in the range from 600 to 700 nm for Cu^2+^ complexes.^52,53^ Based on the presented spectra, the maximum absorbance for the complex in the precursor liquid solution occurs around 704 nm. However, the crystallization process caused a shift of 93 nm toward longer wavelengths (797 nm). This change, known as a bathochromic shift, can be attributed to enhanced intermolecular interactions in the solid phase. These interactions stabilize the electronic states of the complex, thereby lowering the energy required for the electronic transitions and causing a shift to higher wavelengths. Additionally, the spectrum measured in the crystal shows a significantly broader band compared to that of the sample in the precursor liquid solution. Specifically, when Cu^2+^ (3d^9^) occupies a regular octahedral site within the coordination compound, its 5d orbitals split into three lower-energy orbitals and two higher-energy degenerate orbitals. As for the case of Cu^2+^ coordinated by l-histidine and Cl^–^ ions, the octahedral geometry is distorted,^28^ resulting in unequal bond lengths between Cu^2+^ and its ligands along the *z*-axis and in the equatorial plane. This distortion removes the degeneracy of the high-energy orbitals, causing splitting of the d–d transition band, which is reflected as a broad absorption band in the UV–vis spectrum. In many Cu^2+^ complexes, this phenomenon is typically attributed to the Jahn–Teller effect.^54−56^ However, in the case of the [Cu(l-His)Cl_2_] complex, the distortion likely arises from the geometry and nature of the Cl, O, and N ligands, which can influence shaping the structure of the complex.

Additionally, UV–vis absorption measurements were performed to monitor the stability of the complex in solution over time, as shown in Figure 1b. For this test, a 200 mM concentration of the crystal powder was dissolved in 20 mL of a water–methanol mixture under constant magnetic stirring at 30 rpm. The pH and conductivity of the solution were also monitored throughout the experiment. As shown in the spectra, the complex remained stable over 48 h, with the pH showing a slight variation between 7.0 and 7.1 and conductivity remaining steady at approximately −107.3 mV.

### 3.2 XRPD at Room Temperature and Optimized Geometry Analysis

**Figure 2:**
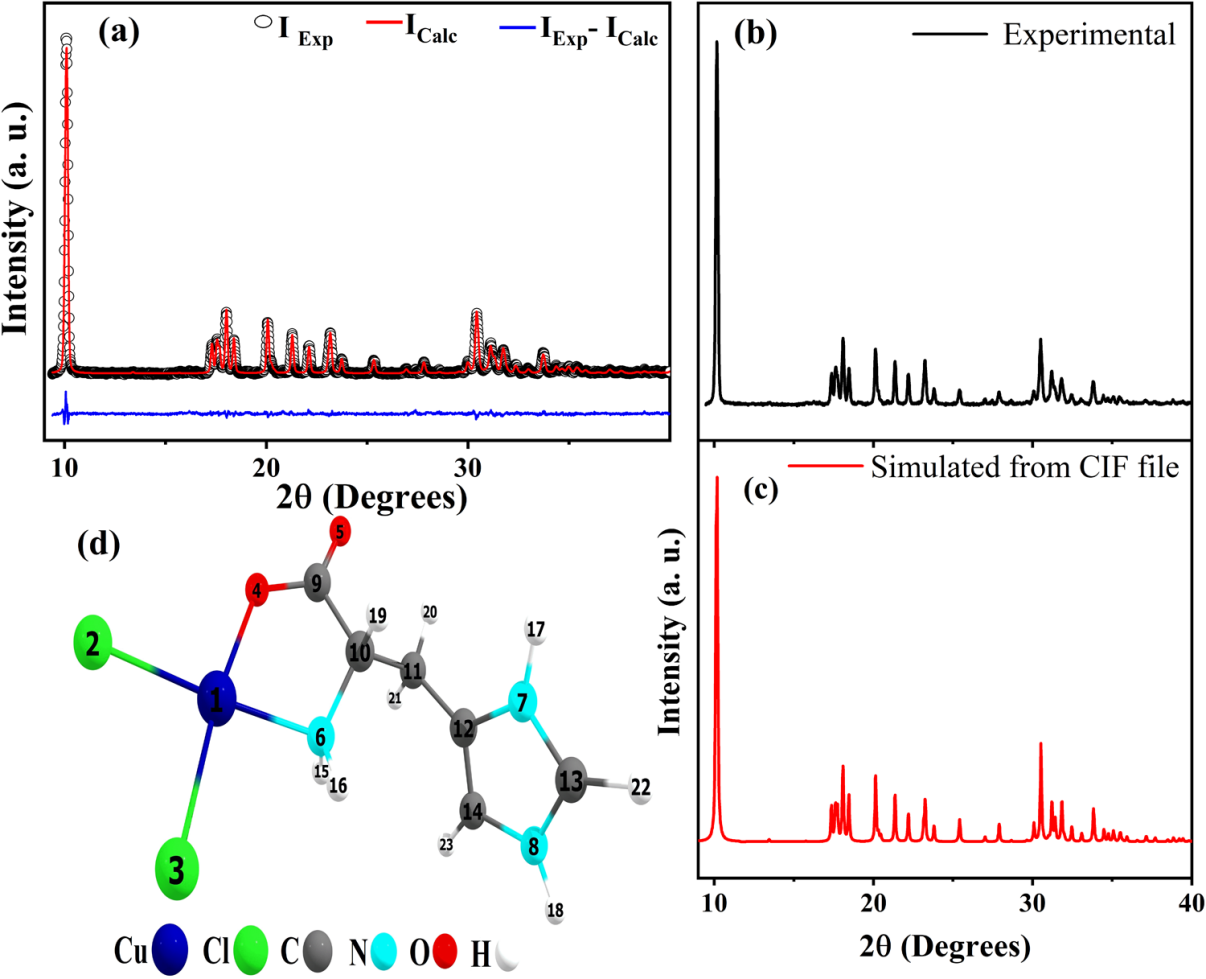
(a) Rietveld’s refinement of XRPD pattern [Cu(l-His)Cl_2_] crystal at room temperature. (b) Experimental XRPD pattern, (c) simulated diffractogram from the crystal CIF file, and (d) optimized geometry of the [Cu(l-His)Cl_2_] complex.

Figure 2a shows the experimental (points) and calculated (red line) [Cu(l-His)Cl_2_] XRPD patterns. The R-factors estimated from Rietveld’s refinement analysis are *R*_wp_ = 4.42% and *R*_p_ = 3.64%, with a 1.1 value obtained for the goodness of fit indicator (S). These values indicate the reliability of the refinement results for the [Cu(l-His)Cl_2_] complex.^57^ The complex crystallizes in the orthorhombic structure (*P*2_1_2_1_2_1_-space group) at room temperature (300 K), with four metallic centers per unit cell (*Z* = 4). The obtained lattice parameters are *a* = 9.992 (7) Å, *b* = 5.937 (9) Å, and *c* = 17.472 (6) Å. Figure 2b,c compares the experimental data with the simulated pattern from the .cif file deposited in CCDC (2373288), where the similarity between the patterns can be observed.

The full optimized geometry of the [Cu(l-His)Cl_2_] complex was obtained through calculations employing the DFT functional PBE1PBE, which is in good agreement with experimental data, like in previous studies of complexes developed with the same functional.^58,59^ In these calculations, a 6-311++G(d,p) basis set was employed for Cl, C, N, and O atoms, while the Stuttgart-Dresden pseudopotential and SDD basis set were utilized for the Cu atom. Additionally, solvation effects in methanol were accounted for using the IEFPCM method. The total charge assigned to the system in the calculations for the complex is 0, and the spin multiplicity is 2. The optimized geometry in methanol is shown in Figure 2d, which was confirmed as the geometry corresponding to a minimum in the potential energy surface since all vibrational frequencies calculated are positive.

In the optimized geometry, the l-His molecule binds to Cu^2+^ through the oxygen atom of the carboxylate and the nitrogen atom of the amine group, consistent with findings from previous studies.^19,24^ The results indicated that the geometry of the complex corresponds to distorted square planar- arrangement around the [Cu(l-His)Cl_2_] complex’s Cu^2+^ ions. This observation aligns with the versatility of Cu^2+^, which can adopt various geometries such as distorted octahedral, square planar, and square pyramidal, depending on the coordinating ligands.^60^ Furthermore, the imidazole ring maintains a flat conformation in the optimized geometry but is bent relative to the planar square formed by the atoms around the Cu^2+^. This structural feature is observed in the N6–C10–C11–C12 dihedral angle.

Table 1 gives the relaxed bond lengths, bond angles, and dihedral angles for the [Cu(l-His)Cl_2_] complex calculated using DFT. In general, calculated geometric parameters exhibit good agreement with the experimental results,^28^ with minor discrepancies related to the dihedral angles. These differences may be associated with the absence of intermolecular interactions in our simulations, which are present in the crystalline environment of the sample. Specifically, the calculated Cu1–O4 bond length is shorter than the Cu1–N6 bond length.

**Table 1:** Geometric Parameters of [Cu(l-His)Cl_2_] Complex: Bond Lengths (Å), Bond Angles (deg), and Dihedral Angles (deg) Calculated (cal.) Using the DFT Functional PBE1PBE

bond lengths (Å)		bond angles (deg)		dihedral angles (deg)	
bond	cal.	angle	cal.	angle	cal.
Cu1–Cl2	2.260	Cl2–Cu1–Cl3	96.714	Cl2–Cu1–O4–C9	172.74
Cu1–Cl3	2.281	Cl2–Cu1–O4	92.362	Cl3–Cu1–O4–C9	15.17
Cu1–O4	1.969	Cl2–Cu1–N6	171.82	N6–Cu1–O4–C9	–12.47
Cu1–N6	2.029	Cl3–Cu1–O4	170.18	Cl2–Cu1–N6–C10	63.07
O4–C9	1.281	Cl3–Cu1–N6	89.972	Cl3–Cu1–N6–C10	–152.02
O5–C9	1.226	O4–Cu1–N6	81.310	O4–Cu1–N6–C10	23.45
N6–C10	1.466	Cu1–O4–C9	117.77	Cu1–O4–C9–O5	–179.23
N7–C12	1.382	Cu1–N6–C10	109.41	Cu1–O4–C9–C10	–1.71
N7–C13	1.327	C12–N7–C13	110.35	Cu1–N6–C10–C9	–29.48
N8–C13	1.325	C13–N8–C14	109.82	Cu1–N6–C10–C11	–155.27
N8–C14	1.373	O4–C9–O5	125.46	C13–N7–C12–C11	178.96
C9–C10	1.541	O4–C9–C10	114.99	C13–N7–C12–C14	0.11
C10–C11	1.531	O5–C9–C10	119.51	C12–N7–C13–N8	–0.14
C11–C12	1.486	N6–C10–C9	108.59	C14–N8–C13–N7	0.18
C12–C16	1.362	N6–C10–C11	114.36	C13–N8–C14–C12	–0.05
		C9–C10–C11	111.85	O4–C9–C12–N6	21.28
		C10–C11–C12	113.85	O4–C9–C10–C11	148.51
		N7–C12–C11	123.52	O5–C9–C10–N6	–161.05
		N7–C12–C14	105.45	O5–C9–C10–C11	–33.82
		C11–C12–C14	131.02	N6–C10–C11–C12	–64.25
		N7–C13–N8	107.26	C9–C10–C11–C12	–171.62
		N8–C14–C12	107.12	C10–C11–C12–N7	–76.44
				C10–C11–C12–C14	102.10
				N7–C12–C14–N8	–0.03
				C11–C12–C14–N8	–178.77

The calculated values of variation of Gibbs free energy (Δ*G*^298^), enthalpy (Δ*H*), and zero-point vibrational energy corrected with zero-point vibrational energy (Δ*E*_ZPVE_) of the complexation in methanol were −191.96, −216.84, and −215.46 kcal/mol, respectively. These negative values of Δ*G*^298^ and Δ*H* indicate that the formation of the complex is spontaneous and exothermic. The value of Δ*G*^298^ also corroborates the experimental TG and DTA findings in the thermal study discussed in detail in Section 3.6.

### 3.3 Chemical Reactivity Descriptors

**Figure 3:**
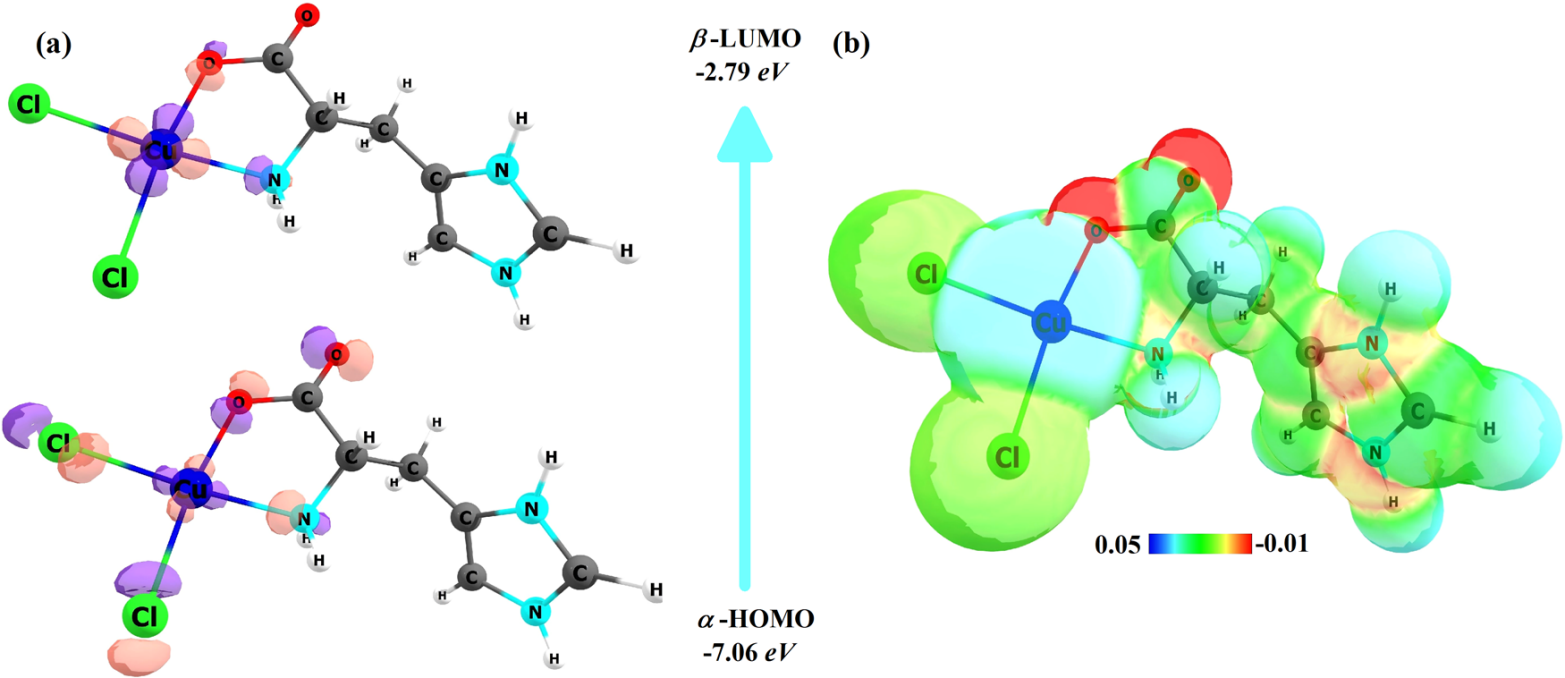
(a) HOMO and LUMO plot and (b) electrostatic potential map of the [Cu(l-His)Cl_2_] complex.

HOMO and LUMO energy values are important parameters for studying the electronic properties of a molecular system. Figure 3a shows the HOMO and LUMO isosurfaces and their respective energy values. HOMO is located mainly on chlorine and copper atoms, but is also over the COO and NH_2_ groups of the His ligand. LUMO is more concentrated on copper Cu^2+^ and oxygen and nitrogen atoms of His. From the energy values of the HOMO and LUMO orbitals, it was possible to calculate several other parameters: HOMO–LUMO energy gap (HLG), chemical potential, electronegativity, hardness, softness, and electrophilicity.^61,62^ These values are listed in Table 2.

**Table 2:** Global Reactivity Descriptors of the [Cu(l-His)Cl_2_] Complex Using the PBE1PBE Functional

quantum chemical descriptor	energy
α-HOMO	–7.06 eV
β-LUMO	–2.79 eV
ionization energy (*I*)	7.06 eV
electronic affinity (*A*)	2.79 eV
energy gap (HLG)	4.27 eV
electronegativity (χ)	4.93 eV
chemical potential (μ)	–4.93 eV
hardness (η)	2.14 eV
softness (σ)	0.47 eV^–1^
electrophilicity index (ω)	5.68 eV

In a theoretical study, it is important to establish correlations between the reactivity parameters and the biological activity of the complexes. For example, a notable correlation is observed between the global electronegativity of a system and its biological activity. A lower global electronegativity indicates greater electron freedom within the system, eventually enhancing its bioactivity. In this study, the [Cu(l-His)Cl_2_] complex exhibits a relatively small global electronegativity of 4.93 eV. It means that the electrons exhibit increased mobility, facilitating their binding to the acceptor sites of the biomolecule.^63^ Also, the HLG value of 4.27 eV indicates that the electronic structure of the [Cu(l-His)Cl_2_] complex is stable, with a small probability of electronic transitions occurring in the complex.^64^

The [Cu(l-His)Cl_2_] complex is an open-shell system with a *d*^9^ configuration and an unpaired electron possessing an inherent magnetic moment that can interact with the external magnetic fields. Furthermore, the presence of this unpaired electron can induce distortions in the complex molecular geometry due to electrostatic repulsion between neighboring ions. To thoroughly investigate such systems, it is essential to employ separate spatial orbitals for α and β electrons, as was adopted in the present study.^59^

In Figure 3b, the electrostatic potential map of the [Cu(l-His)Cl_2_] complex highlights regions with varying electron densities: those in red have higher electron density, while those in blue represent areas with lower electron density. The green regions correspond to sites with an average electron density. Notably, the O and N atoms of the imidazole ring within the [Cu(l-His)Cl_2_] complex exhibit heightened electron density, while the Cu and H atoms display lower electron density.

### 3.4 Hirshfeld Surface Analysis

**Figure 4:**
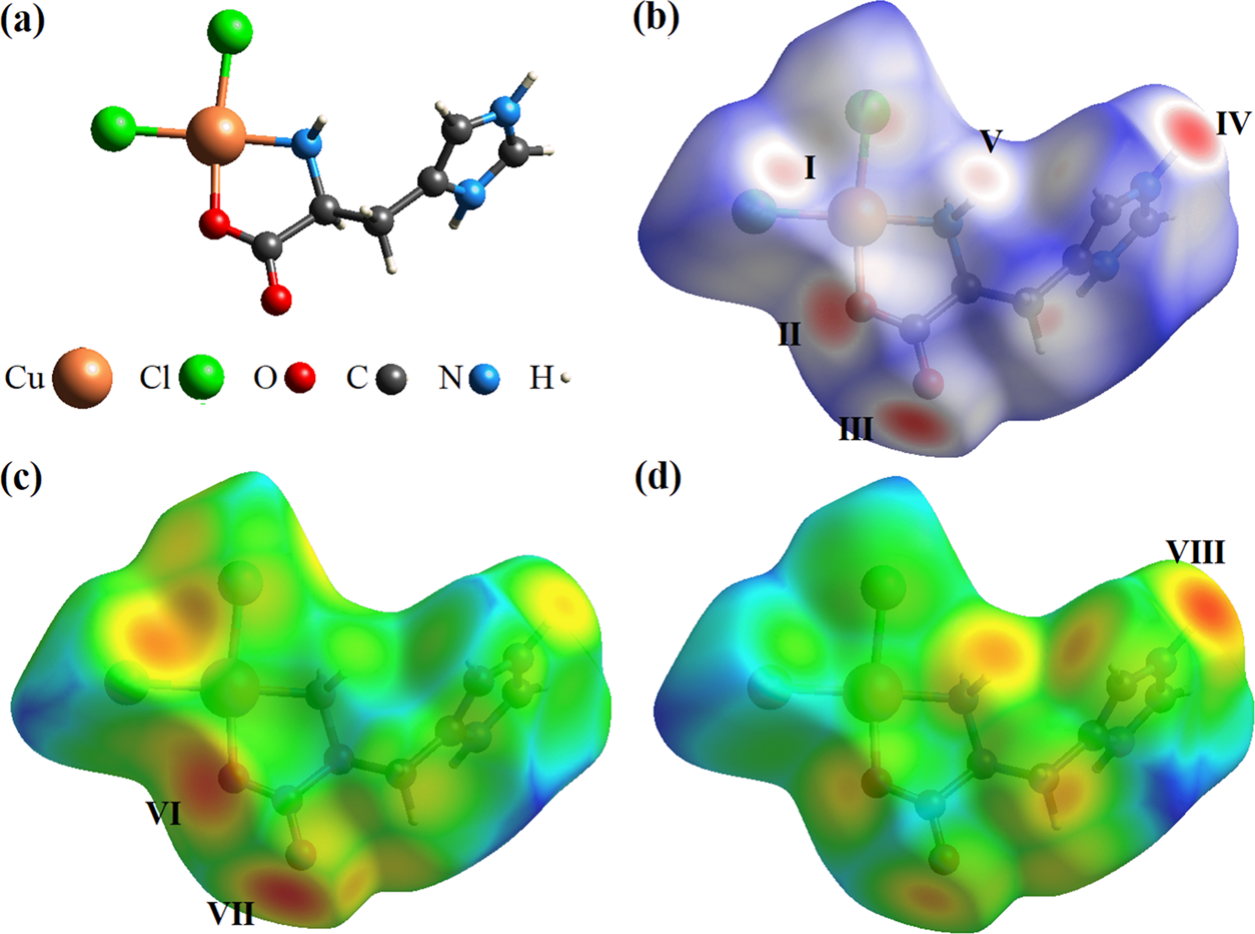
(a) Chemical structure of the [Cu(l-His)Cl_2_] complex and Hirshfeld surfaces as a function of (b) *d*_norm_, (c) *d*_e_, and (d) *d*_i_.

Figure 4 illustrates the chemical structure (a) and 3D Hirshfeld surfaces as functions of *d*_norm_ (b), *d*_e_ (c), and *d*_i_ (d) of the [Cu(l-His)Cl_2_] complex, respectively. In these surfaces, the red regions indicate strong interactions between the closest atoms on the *d*_norm_ surface. Besides, on the *d*_e_ surface, the red color represents receptor regions, while on the *d*_i_ surface, it denotes donor regions. The interaction between the Cl atom and the H atom of the amine group (Cu–Cl···H–N) occurs in the sites indicated by I and V. Furthermore, in the region II occurs interaction between O atoms of the amine group with H of the imidazole ring (C–O···H–C). Region III denotes interactions between the O atom and H atoms of the imidazole ring (C=O···H–N), with the O atom also interacting with the H atom attached to the carbon of the chain (C=O···H–C). In region IV, the H atom interacts simultaneously with the Cl atom (Cu–Cl···H–N) and with the O atom of the carboxyl unit (C–O···H–N), indicated in region III. Interactions II and III (Figure 4b) correspond to regions VI and VII (Figure 4c), respectively. In addition, region IV is equivalent to region VIII of the *d*_i_ map (Figure 4d), indicating the donor region of the intermolecular interaction.

**Figure 5:**
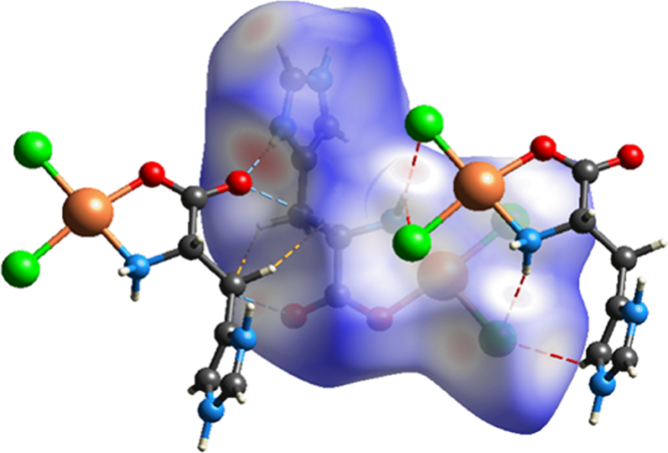
Intermolecular interactions of the [Cu(l-His)Cl_2_] complex: dashed red line (Cl···H), dashed blue line (O···H), and dashed yellow line (H···H).

**Figure 6:**
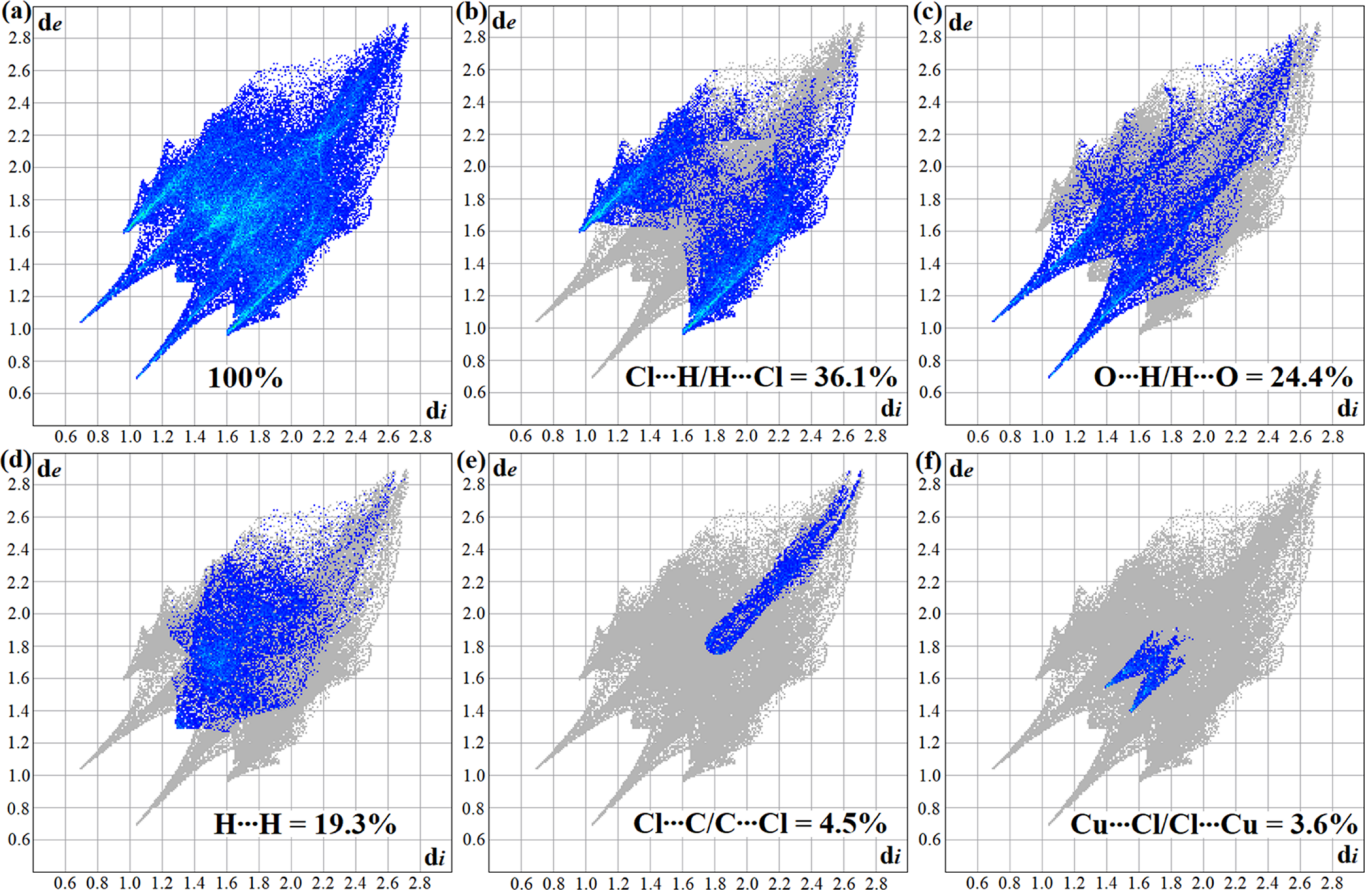
2D fingerprint plots of the [Cu(l-His)Cl_2_] complex: (a) all interactions, (b) Cl···H/H···Cl interactions, (c) O···H/H···O interactions, (d) H···H interactions, (e) Cl···C/C···Cl interactions, and (f) Cu···Cl/Cl···Cu interactions.

Figure 5 provides a detailed model of the intermolecular contacts, highlighting the Cl···H interactions in red, the O···H interactions in blue, and the H···H interactions in yellow. 2D impression plots were utilized to further elucidate these interactions, as shown in Figure 6a–f. The most significant individual contribution found is Cl···H/H···Cl, which accounted for 36.1% of the total interactions (Figure 6b). The O···H/H···O (Figure 6c) and H···H (Figure 6d) contacts were significant in the [Cu(l-His)Cl_2_] complex, constituting 24.4 and 19.3% of the total interactions, respectively.

The observed peaks in the Cl···H/H···Cl and O···H/H···O interactions at low *d*_e_ + *d*_i_ values indicate their strength. While Cl···C/C···Cl (Figure 6e) and Cu···Cl/Cl···Cu (Figure 6f) contacts were also observed, their contributions to the overall surface were comparatively lesser. The results from the 2D impression plots show the prevalence of hydrogen bonding as the primary intermolecular interaction, confirming their substantial contributions to the stability of the crystal.

### 3.5 Group Theory and Vibrational Analyses

As previously mentioned, the crystal structure of the [Cu(l-His)Cl_2_] complex exhibits orthorhombic symmetry (*P*2_1_2_1_2_1_ (*D*_2_^4^) space group), with four coordination units per unit cell and 23 atoms in each coordination. Consequently, the unit cell consists of 92 atoms. According to group theory,^65^ the irreducible representation of IR and Raman-active modes related to the *D*_2_ factor group is given by Γ = 3A + 3B_1_ + 3B_2_ + 3B_3_. Considering the 23 occupied sites within the [Cu(l-His)Cl_2_] unit cell, the total representation of the normal vibration modes in the irreducible representations is Γ_total_ = 69A + 69B_1_ + 69B_2_ + 69B_3_. However, the acoustic modes are characterized by Γ_ac_ = 1B_1_+ 1B_2_ + 1B_3_. Therefore, IR-active modes are described by Γ_IR_ = 68B_1_ + 68B_2_ + 68B_3_, and Raman-active modes are denoted by Γ_Raman_ = 69A + 68B_1_ + 68B_2_ + 68B_3_.

**Figure 7:**
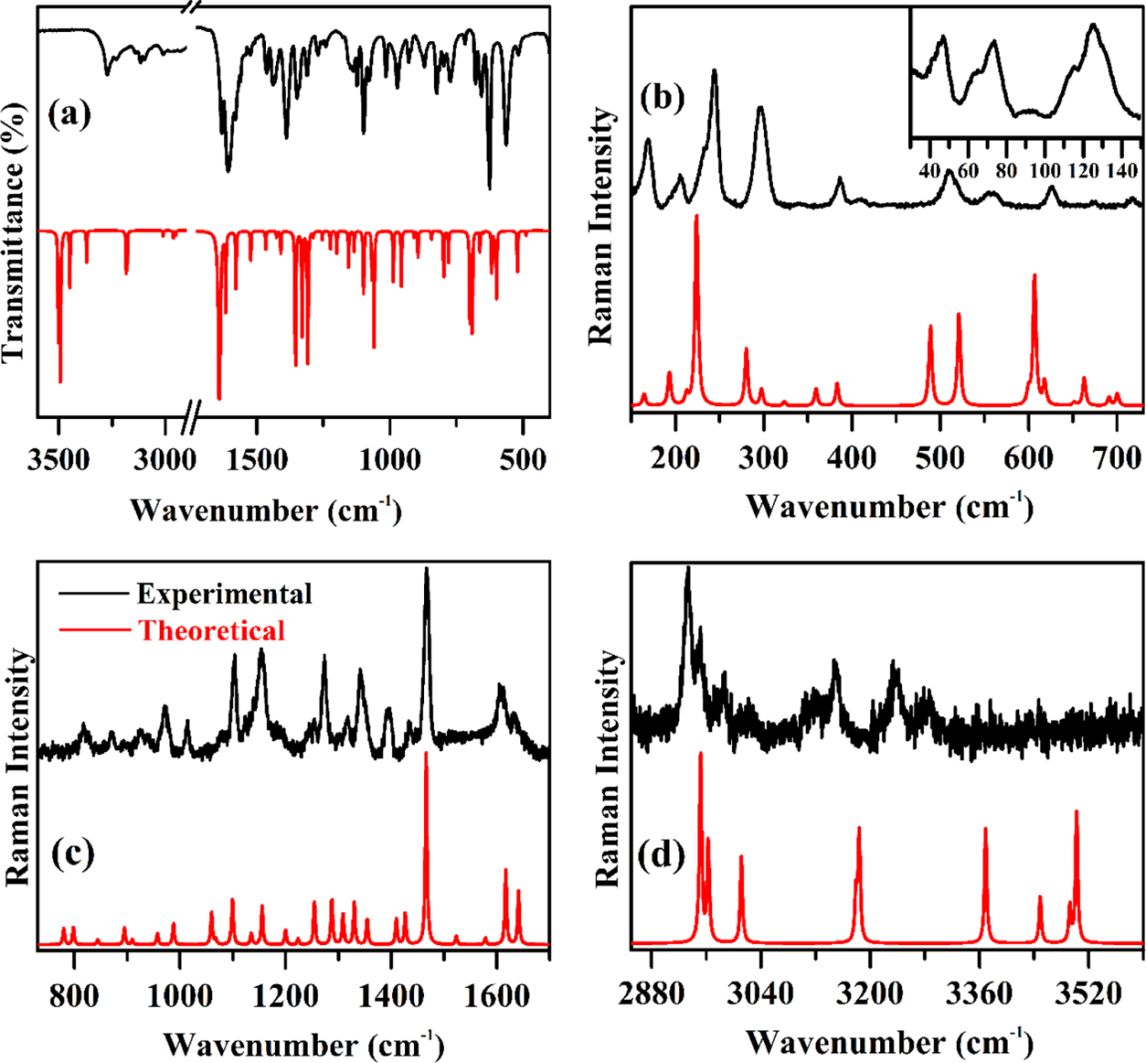
(a) Experimental (black) and theoretical (red) FT-IR spectra of the [Cu(l-His)Cl_2_] crystal in the spectral range from 3600 to 300 cm^–1^. Experimental (black) and theoretical (red) Raman spectra of the [Cu(l-His)Cl_2_] complex in the spectral ranges of (b) 150–730 cm^–1^, (c) 730–1700 cm^–1^, and (d) 2850–3600 cm^–1^. The inset in (b) shows the 30–150 cm^–1^ range.

Experimental and simulated FT-IR and Raman spectra are shown in Figure 7a–d. Table 3 provides the peak positions of the FT-IR and Raman bands and their corresponding vibrational assignments. Notably, the experimental modes exhibit good agreement with the calculated vibration modes.

**Table 3:** Assignments (Experimental and Calculated) of Vibration Modes (cm^–1^) for the [Cu(l-His)Cl_2_] Complex with VMARD ≥9%, Observed Raman Band Position (ω_Raman_), Experimental IR Band Position (ω_IR_), and Calculated Band Position (ω_calc_)

ω_Raman_	ω_IR_	ω_calc_	attributions using VMARD^36^ (%)^a^
		3502	ν(N7H17) (23%) + ν(N8H18) (59%)
		3493	ν(N7H17) (56%) + ν(N8H18) (23%)
		3448	ν_a_(H15N6H16) (93%)
		3369	ν_s_(H15N6H16) (94%)
3286	3273	3184	ν (C13H22) (57%) + ν (C14H23) (18%)
3236	3228	3178	ν(C14H23) (60%) + ν(C13H22) (20%)
3149	3142	3011	ν_a_(H20C11H21) (88%)
3116	3098	2963	ν_s_(H20C11H21) (73%) + ν(C10H19) (21%)
2986	3011	2951	ν(C10H19) (57%) + ν_s_(H20C11H21) (37%)
2949		1640	ν_a_(O4C9O5) (80%)
2933		1617	δ ring (56%) + ν_a_(C11C12C14) (24%)
1632	1632	1578	δ_s_(H15N6H16) (36%) + δ(C10N6H15H16) (21%)
1609	1577	1523	δ ring (78%)
	1526	1466	δ ring (76%)
	1508	1427	ν_a_(C12N7C13) (25%) + δ ring (22%) + ν(C12C14) (9%)
1467	1464	1409	δ_s_(H20C11H21) (14%) + ν(C12C14) (11%) + ν(N7C13) (9%)
1434	1431	1355	ν_s_ (O4C9O5) (26%) + δs (N6C10H19) (15%)
1395	1390	1330	ν_s_(O4C9O5) (28%) + δ_a_(C11C10H19) (9%)
1342	1358	1308	ν_s_(O4C9O5) (32%)
1317	1338	1288	ν_a_(C11C12N7) (21%)
1300	1309	1254	ν_s_ ring (47%)
1272	1269	1222	ν_s_(C12N7C13) (18%) + ν_s_(C14N8C13) (14%) + δ_a_(C10C11C12) (10%)
1255	1240	1200	δ_s_(C9C10H19) (9%) + ν_s_(O4C9O5) (14%)
1244		1156	ν(N8C13) (30%) + δ_a_(C12N7C13) (10%)
1192	1178	1135	ν_a_(C12N7C13) (31%) + δ_s_(H17N7C13H22) (29%)
1155	1148	1099	ν_a_(N6C10C11) (29%)
1141	1123	1067	ν(N8C14) (29%) + ν_a_(N8C13N7) (19%) + δ_a_(N8C14H23) (11%) + δ_s_(H22C13N8H18) (9%)
1124	1098	1059	ν_a_(O4C9C10) (13%)
1014	1014	988	δ_s_(C12N7C14) (16%)
971	972	957	δ_s_ ring (31%) + ν(C10N6C11) (12%)
938		910	δ ring (69%)
926	929	895	ν_s_(O4C9O5) (20%) + ν(C9C10) (9%)
871	870	844	ν(N6C10) (10%) + τ(C13N7C12C11) (14%)
827		798	δ_s_(O4C9O5) (12%)
816		779	ν_a_(O4C9O5) (11%)
		701	δ_a_(C10C11C12) (9%) + δ_a_(N7C12C14) (9%)
717		692	τ ring (21%)
		663	δ_a_(N7C12C14) (10%) + ν(C11C12) (10%)
674		651	τ(H17N7C13N8) (9%)
	628	618	δ_a_(Cu1N6H15) (10%)
626	562	607	ν(C9C10) (10%)
558	514	600	τ(H17N7C13N8) (15%) + γ_s_(C12N7C13H17) (13%) + τ(N7C13N8C14) (10%) + τ(C13N7C12C11) (10%)
510		521	δ_a_(Cu1N6C10) (14%) + ν(C9C10) (13%) + δ_α_(O5C9C10) (12%) + ν(O5C9) (9%)
410		488	ν(Cu1N6) (14%) + δ_a_(O5C9C10) (14%) + δ_a_(Cu1O4C9) (11%)
386		383	ν(Cu1O4) (12%)
		359	ν_a_(Cl2Cu1N6) (13%)
		323	δ_a_(N7C12C11) (9%)
		297	ν_s_(Cl2Cu1Cl3) (38%) + ν(Cu1O4) (10%) + δ_a_(O4Cu1N6) (9%)
296		281	ν(Cu1Cl2) (17%)
244		224	ν(Cu1Cl3) (17%) + ν(Cu1O4) (14%) + δ_a_(Cu1O4C9) (13%) + ν(O4C9) (9%)
233		213	δ_a_(O5C9C10) (11%) + δ_a_(C9C10C11) (11%)
206		193	τ(N6Cu1Cl2) (20%)
169			lattice
125			
115			
92			
73			
64			
46			
40			

a τ = torsion; ν = stretching; ν_a_ = antisymmetric stretching; ν_s_ = symmetric stretching; δ_a_ = antisymmetric bending; δ = symmetric bending.

In the 3502–2950 cm^–1^ range, both calculated and experimental IR absorption bands are attributed to stretching vibrations of the NH_2_, NH, CH, and CH_2_ groups.^66^ The vibration modes observed at high wavenumbers in the Raman spectrum showed the same assignments as those observed in the FT-IR spectrum. Specifically, Raman bands centered at 3286, 3236, and 2986 cm^–1^ correspond to stretching of the C13H22, C14H23, and C10H19 bonds. The vibration modes observed at about 3149 and 3116 cm^–1^ denote the antisymmetric and symmetric stretching of the CH_2_ group, respectively. The Raman bands observed at about 1434, 1395, 1342, 1255, 938, and 827 cm^–1^ are mainly associated with stretching and deformation of the COO group. The observed shift in bands related to the COO group may be attributed to the intermolecular hydrogen bonds.

In the Raman spectrum, vibration bands attributable to lattice modes (30–150 cm^–1^) are shown in Figure 7b, inset. Additionally, the region between 30 and 200 cm^–1^ presents the bands related to the crystalline lattice vibrational modes that can couple to the motions from intermolecular hydrogen bonds.^67,68^ Moreover, bands related to metal–ligand interactions are noticeable in the region between 200 and 450 cm^–1^. Specifically, the Raman band centered at 510 cm^–1^ is associated with an asymmetric bond deformation (Cu1N6C10), and the Raman bands located at about 410, 386, 296, and 244 cm^–1^ are mainly assigned as stretching modes from the Cu1N6, Cu1O4, CuCl12, and CuCl13 bonds, respectively. The vibration modes observed between 670 and 830 cm^–1^ are related to the motions from carbon–oxygen bonds in the carboxyl and nitrogen–carbon bonds of the imidazole ring. Overall, the Raman and FT-IR spectra results provide complementary information, with lower-wavenumber vibration bands being more prominent in the Raman spectrum. Moreover, a noteworthy observation is the close agreement between theoretical and experimental vibration bands regarding their profiles and positions.

### 3.6 Thermal Analyses and XRPD at High-Temperature Conditions

**Figure 8:**
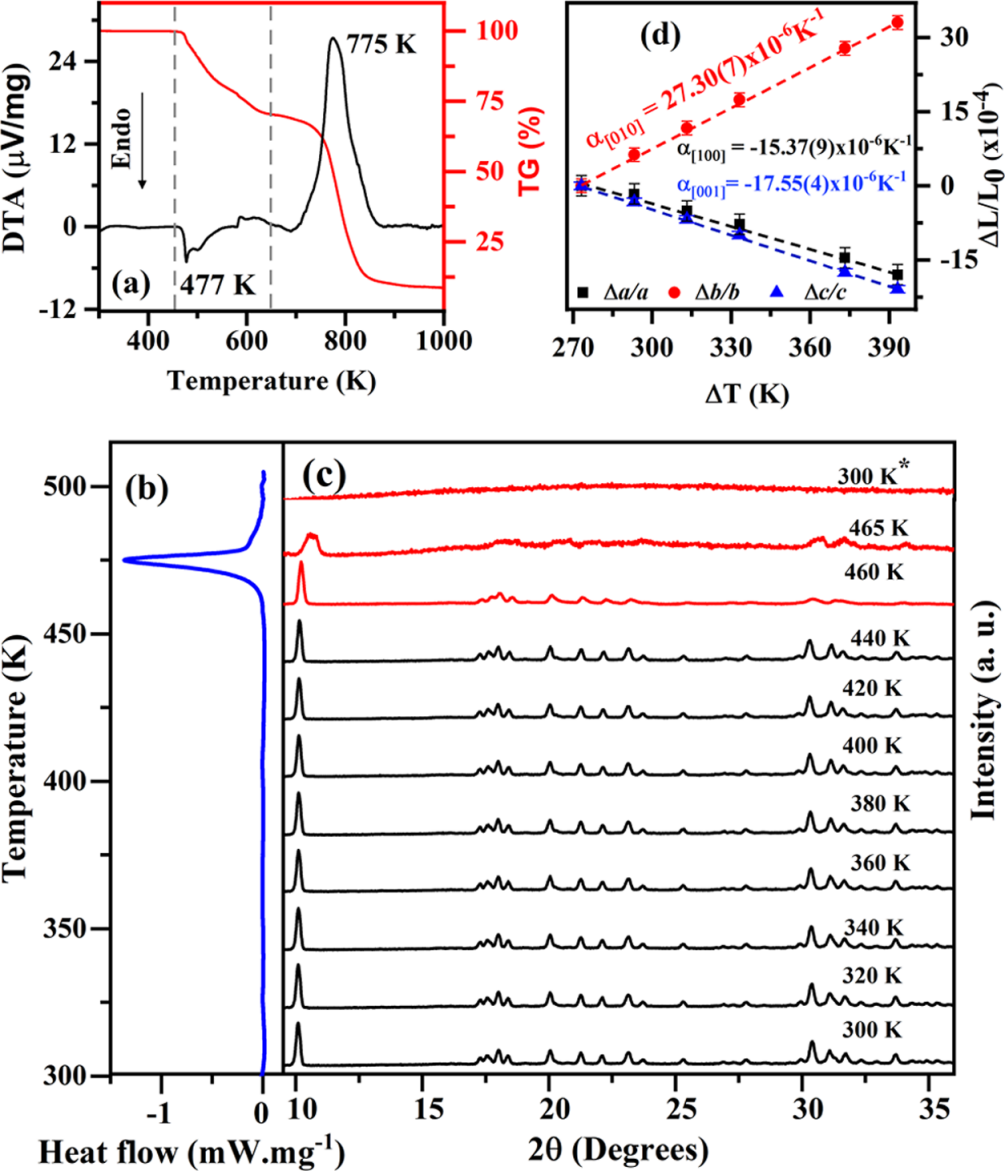
(a) TG and DTA curves and (b) DSC curve of the [Cu(l-His)Cl_2_] powdered sample. (c) XRPD patterns of the [Cu(l-His)Cl_2_] powdered sample as a function of temperature in the interval from 300 to 465 K. The * symbol indicates the measurements performed after cooling the sample to 300 K. (d) Thermal expansion coefficient of the [Cu(l-His)Cl_2_] crystal up to 400 K.

The TG, DTA, and DSC thermal analyses of the [Cu(l-His)Cl_2_] powdered sample are shown in Figure 8a,b, respectively. The TG curve exhibits two distinct events corresponding to mass loss in the sample. The first thermal event occurs between 460 and 636 K, resulting in a molar mass loss of 84.50 g·mol^–1^, associated with the initial decomposition of His and the release of chloride ions. Subsequently, the second event, observed in the 689–860 K interval, results in a mass loss close to 189.2 g·mol^–1^, corresponding to the total decomposition of His. The final residual mass is associated with copper in its oxide form.

In the DTA curve, an endothermic peak is observed within the 450–700 K range, indicating the melting and decomposition of the [Cu(l-His)Cl_2_] complex. Additionally, an exothermic peak at around 775 K suggests the oxidative degradation process of the organic part during the decomposition of the complex, similar to the thermal behavior observed in bis (l-threonine) Cu^2+^ monohydrate crystal previously reported in the literature.^69^ The DSC curve displays a single endothermic peak at approximately 477 K associated with the [Cu(l-His)Cl_2_] complex melting, indicating its robust thermal stability from room temperature up to ∼447 K.

Figure 8c displays XRPD patterns of [Cu(l-His)Cl_2_] at elevated temperatures ranging from 300 to 465 K. The orthorhombic crystallographic arrangement of [Cu(l-His)Cl_2_] remains stable up to 440 K. However, as the temperature rises beyond this point, there is a gradual broadening and reduction in the intensity of XRPD peaks. Notably, the well-defined peaks located at 2θ = 10.10, 30.42, 31.08, and 31.70° are no longer observed in the pattern measured at *T* = 465 K, evidencing a continuous process of the crystallographic order loss. The subsequent measurement after the cooling-down sample from 465 to 300 K, denoted by 300 K*, does not exhibit a recovery of the orthorhombic structure peaks. This observation suggests a gradual and irreversible transformation from an orthorhombic crystalline structure to an amorphous phase, consistent with the findings from thermal analysis.

From the refined parameters of the [Cu(l-His)Cl_2_] crystal unit cell as a function of temperature, it was possible to calculate the crystal’s thermal expansion coefficients within the temperature range of 300 to 400 K. Figure 8d shows the temperature dependence variation of the normalized lattice parameters, Δ*L*/*L*_0_ (with respect to their values at *T* = 300 K). Linear thermal expansion coefficients (α_[hkl]_) were calculated from linear fitting of the Δ*L*/*L*_0_ data as a function of temperature. The obtained slopes values are as follows: α_[100]_ = −15.37(9) × 10^–6^ K^–1^, α_[010]_ = 27.30(7) × 10^–6^ K^–1^ and α_[001]_ = −17.55(4) × 10^–6^ K^–1^. These results indicate negative thermal expansion along the *a* and *c* axes, whereas the *b* axis exhibits positive thermal expansion. Such behavior suggests anisotropy in the [Cu(l-His)Cl_2_] complex unit cell under high-temperature conditions, which can be related to the different spatial orientations of hydrogen bonds within the crystal, as similarly occurred in the l-histidinium bromide monohydrate crystals.^70^

### 3.7 Antimicrobial Activity (AA)

Table 4 displays the antimicrobial test results assessing the minimum inhibitory concentration (MIC) of the [Cu(l-His)Cl_2_] complex. Additionally, it includes the data obtained for the standard drugs Meropenem and fluconazole control samples. The [Cu(l-His)Cl_2_] complex demonstrated potent antimicrobial activity against the gram-positive bacterium *S. aureus*, with an MIC of 1.5 μM. Conversely, the complex did not exhibit efficient activity against the gram-negative bacterium *P. aeruginosa*, with an MIC greater than 200 μM. Notably, the standard drug Meropenem^71,72^ tested as a control sample demonstrated a lower MIC (130 μM) against *S. aureus* compared with the [Cu(l-His)Cl_2_] complex.

**Table 4:** Minimal Inhibitory Concentration (MIC) of the [Cu(l-His)Cl_2_] Crystal and Control Drugs Meropenem and Fluconazole against the Bacteria *S. aureus* and *P. aeruginosa* and *C. albicans* Fungus

type of microorganism	microorganism	[Cu(l-His)Cl_2_] (μM)	meropenem (μM)	fluconazole (μM)
Gram-positive bacteria	*Staphylococcus aureus*	1.5	130	
Gram-negative bacteria	*Pseudomonas aeruginosa*	>200	130	
fungus	*Candida albicans*	50		13

Several copper complexes reported in the literature exhibit remarkable biological activity against specific microorganisms. In fact, studies have shown the DNA binding ability of the Cu^2+^ complexes.^73−75^ The complex chemical structure can be essential to this process. Specifically, in the case of the [Cu(l-His)Cl_2_] complex, the l-his ligand can facilitate the transport of Cu^2+^ ions across the cell membrane, enhancing their penetration and increasing membrane permeability. Additionally, the chlorine atom can enhance the reactivity of the molecule, leading to oxidative damage in the pathogenic microorganism.

Comparing this study with other Cu(II) complexes reported in the literature, one can note that the complex is among the most potent Cu(II) complexes against Gram-positive bacterium *S. aureus*, which reported MIC values between 0.4 and 3 μM. Similar MIC values have been reported for other Cu(II) compounds, including the pyrithione complexes [Cu(PyS)_2_] and [Cu(PyS1)_2_], where PyS refers to pyrithione, and PyS1 is a modified version with a shorter polyethylene glycol chain.^76^ Other examples include CuCl_2_·2H_2_O^77^ and several Cu(II) norfloxacin (NFL) complexes.^77^ Additionally, Cu(II) complexes with enrofloxacin (erx) and various bidentate ligands, such as [Cu(erx)(2,9-dimethyl-1,10-phenanthroline)Cl] and [Cu(erx)(5-nitro-1,10-phenanthroline)Cl], have also shown comparable activity.^78^

Furthermore, the [Cu(l-His)Cl_2_] complex exhibited an MIC of 50 μM against the fungus *Candida albicans*, indicating reduced activity compared to the fluconazole control sample, which presented an MIC of 13 μM. These findings suggest that the formation of the [Cu(l-His)Cl_2_] complex did not substantially enhance antimicrobial activity against both *C. albicans* and *P. aeruginosa*. It can be inferred that the antimicrobial efficacy of the [Cu(l-His)Cl_2_] complex is influenced by the structure of the microorganism’s cell wall.

The reduced efficacy of the [Cu(l-His)Cl_2_] complex against Gram-negative *P. aeruginosa* bacteria may be related to the complex composition of their cell wall. Gram-negative bacteria possess a multilayered cell wall with distinct chemical compositions, comprising a layer of peptidoglycan and a secondary layer consisting of lipopolysaccharides and lipoproteins.^79,80^ Moreover, it has an outer membrane that serves as an additional barrier, hindering the penetration of antimicrobial agents.^81^ Consequently, Gram-negative bacteria’s cell wall structure is more intricate than that of Gram-positive bacteria, which, albeit thicker, primarily consists of a single type of macromolecule.^80^

### 3.8 Cell Viability

**Figure 9:**
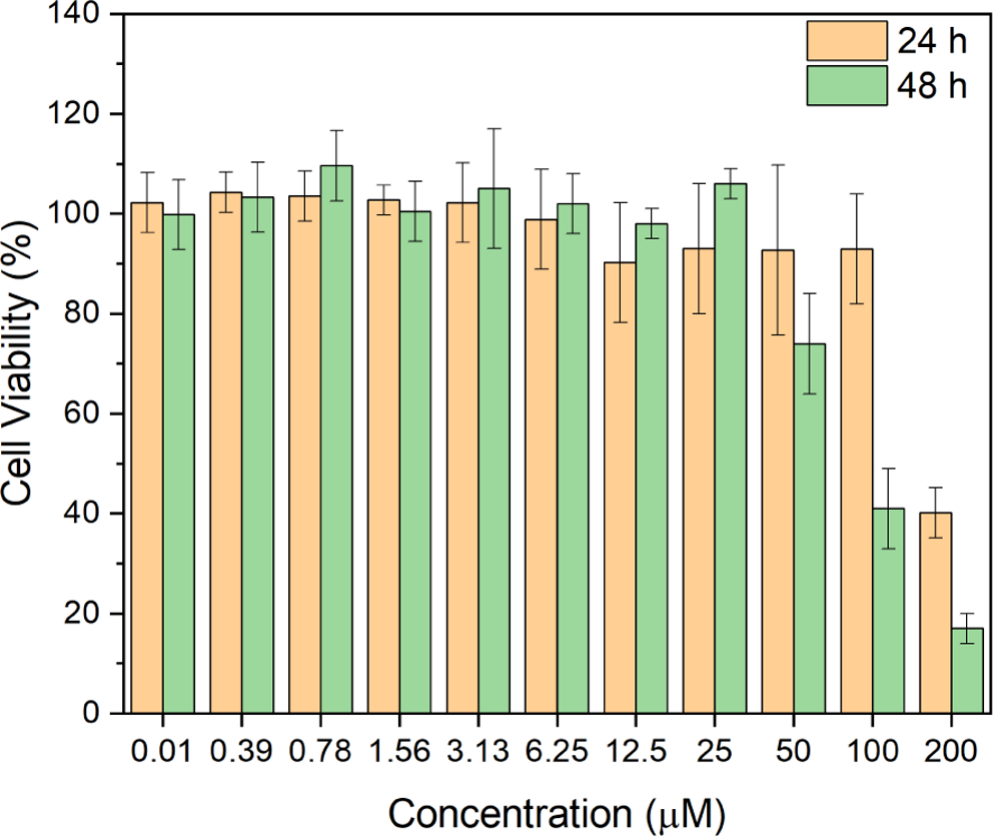
Viability of the human cell line GM07492A after exposure to varying concentrations of the [Cu(l-His)Cl_2_] complex, ranging from 0.39 to 200 μM, for 24 and 48 h.

The inhibitory effects of the [Cu(l-His)Cl_2_] complex on the growth of human cell line GM07492A were assessed using the MTT method. Various concentrations of the [Cu(l-His)Cl_2_] complex (ranging from 0.39 to 200 μM) were utilized to incubate the GM07492A cells for 24 and 48 h, as shown in Figure 9.

The investigation of cell viability using the lung fibroblast GM07492A cell line was conducted to determine the survival capacity of healthy cells in contact with the [Cu(l-His)Cl_2_] complex. Specifically, this cell line has been used as a general model for cytotoxicity screening; if a compound is toxic to these cells, it may also be harmful to other types of normal cells. Such findings would indicate the need for further investigation or reformulation of the compound.

Following 24 h of treatment, the quantity of viable cells remains consistent with the average growth rates observed in the control sample within in vitro culture conditions for the [Cu(l-His)Cl_2_] complex concentrations ranging from 0.39 to 6.25 μM. Noteworthy, no significant variation in cellular viability was detected for concentrations of 12.5, 20, 50, and 100 μM, exhibiting only minor fluctuations within the error margins. However, the highest concentration examined (200 μM) presented a 60% reduction in cell viability. Upon 48 h of incubation, cellular viability exhibited no significant deviation for concentrations ranging from 0.39 to 25 μM when contrasted with control sample metrics. However, at concentrations of 50, 100, and 200 μM, declines in cell growth of 30, 70, and 80%, respectively, were evident, as depicted in Figure 9.

The minimum inhibitory concentration and cell viability tests strongly suggest that the [Cu(l-His)Cl_2_] complex inhibits the growth of *S. aureus* bacteria at a concentration of 1.5 μM while maintaining the viability of the GM07492A cell line without causing damage. This selectivity indicates the [Cu(l-His)Cl_2_] complex’s potential to target *S. aureus* bacteria specifically.

### 3.9 ADME Analysis of Pharmacokinetic Parameters

A critical aspect of drug discovery involves predicting molecular physicochemical parameters, bioavailability, and pharmacokinetics.^82^ It is essential to grasp the molecular descriptors capable of predicting optimal pharmacokinetic properties. ADME (absorption, distribution, metabolism, and excretion)^83^ parameters give insights into bioavailability, lipophilicity, drug similarity, and various physicochemical properties, including water solubility, molecule size, polarity, and flexibility. In this study, ADME calculations were conducted using the SwissADME^47^ online pharmacokinetic platform to assess the potential suitability of the [Cu(l-His)Cl_2_] complex for drug application. The obtained results are listed in Table 5.

**Table 5:** Pharmacokinetic Parameters of the [Cu(l-His)Cl_2_] Complex Obtained from ADME Calculations

Physicochemical Properties	
molecular weight	289.61 g/mol
TPSA^a^	79.54 Å^2^
lipophilicity	
log* P*_o/w_^b^ (SILICOS-IT)	–1.47

Water Solubility	
log *S* (SILICOS-IT)	–1.75
solubility	5.25 mg/mL; 1.78 × 10^–2^ mol/L
class^c^	soluble

Pharmacokinetics	
GI absorption	high
BBB permeant	no
P-gp substrate	yes
CYP1A2 inhibitor	no
CYP2C19 inhibitor	no
CYP2C9 inhibitor	no
CYP2D6 inhibitor	no
CYP3A4 inhibitor	no
log *K*_p_ (skin permeation)	–7.96 cm/s

Druglikeness	
Lipinski^d^	yes; 0 violation
Ghose^e^	no; 1 violation: WLOGP < −0.4
Veber^f^	yes
Egan^g^	yes
Muegge	yes
bioavailability score	0.55

Medicinal Chemistry	
PAINS	0 alert
Brenk	0 alert
leadlikeness	yes
synthetic accessibility	4.20

a Topological polar surface area.

b Partition coefficient between n-octanol/water.

c Insoluble < −10 < poor < −6 < moderately soluble < −4 < soluble < −2 < very soluble < 0 < highly.

d MM ≤ 500; log *P*_o/w_ ≤ 5; H-bond donors ≤5; H-bond acceptors ≤10.

e 180 ≤ MM ≤ 480; 20 ≤ no. of atoms ≤ 70; 40 ≤ molar refractivity ≤130; −0.4 ≤ log *P*_o/w_ ≤ 5.6.

f Num. rotatable bonds ≤10; TPSA ≤ 140 Å^2^.

g log *P*_o/w_ ≤ 5.88; TPSA ≤ 131.6 Å^2^.

The molecular mass of the [Cu(l-His)Cl_2_] complex is 289.6 g/mol, a favorable index for assessing drug similarity according to Lipinski’s rule,^82,84^ in which a molecular mass ≤500 g/mol facilitates the compound permeation into the cell membrane and intracellular access. Regarding the [Cu(l-His)Cl_2_] physicochemical properties, the complex exhibits a topological polar surface area (TPSA) of 79.54 Å^2^. The TPSA values lower than 140 Å^2^ indicate favorable intestinal absorption, while values below 70 Å^2^ indicate good brain penetration. Therefore, pharmacokinetic models project high gastrointestinal (GI) absorption^85,86^ for the [Cu(l-His)Cl_2_] complex.

The log *P*_o/w_ parameter, which measures compound hydrophobicity, is another crucial metric for evaluating drug-like characteristics. The [Cu(l-His)Cl_2_] complex exhibits a log *P*_o/w_ value of −1.47, indicating potential membrane penetration. According to the Lipinski rule,^84^ molecules with values lower than 5 indicate a drug’s similar characteristics.

The obtained value for the water solubility (log *S*) is −1.75, demonstrating the [Cu(l-His)Cl_2_] complex affinity to dissolve in an aqueous medium, indicating solubility. Moreover, it exhibits no inhibition of tested cytochromes (CYP1A2, CYP2C19, CYP2C9, CYP2D6, and CYP3A4), suggesting no risk of hepatotoxicity upon oral intake. The complex demonstrates a value of 0.55 for the bioactivity index, surpassing Lipinski’s biological activity threshold (>0). It corroborates the other obtained parameters. In this context, preliminary *in silico* results unveil a promising pharmacokinetic profile for the [Cu(l-His)Cl_2_] complex.

## 4 Conclusions

In this study, a crystal of the [Cu(l-His)Cl_2_] complex was successfully synthesized using a slow solvent evaporation technique. XRPD results showed that [Cu(l-His)Cl_2_] crystallized in the orthorhombic structure with the *P*2_1_2_1_2_1_ space group. The complex’s optimized geometry, frontier molecular orbitals, electronic properties, and spectroscopic data were obtained by employing DFT calculations. The [Cu(l-His)Cl_2_] complex exhibited a distorted square planar geometry around the Cu^2+^ center. The obtained experimental (FT-IR and Raman) vibrational modes agree with theoretical (DFT) predictions, validating our computational approach. Furthermore, the electron density distribution obtained through electrostatic potential mapping indicated regions of higher electron density around oxygen and nitrogen atoms of the imidazole ring, contrasting with lower-electron-density regions around copper and hydrogen atoms.

Hirshfeld surface analysis revealed hydrogen bonding as the primary intermolecular interaction within the crystal lattice. Additionally, thermal stability analysis demonstrated that the complex remained stable up to 460 K. Above this temperature, the [Cu(l-His)Cl_2_] complex transforms from the ordered orthorhombic arranged to the amorphous phase. Notably, the [Cu(l-His)Cl_2_] complex displayed significant antimicrobial activity against Gram-positive bacterium *Staphylococcus aureus*. Our findings suggest its potential as a therapeutic agent. Minimum inhibitory concentration and cell viability assays indicated that the complex effectively inhibited the growth of *Staphylococcus aureus* bacteria at a concentration of 1.5 μM without causing harm to GM07492A cell lines. The pharmacokinetic parameters corroborate the other tested parameters and highlight the [Cu(l-His)Cl_2_] complex as a promising alternative for future clinical trials and medicinal applications.

## Supporting Information Available

X-ray crystallographic information file obtained by single-crystal (CCDC 2373288) (CIF)

## Supplementary Material

ao4c05029_si_001.cif
